# Hippocampal Neurogenesis and Dendritic Plasticity Support Running-Improved Spatial Learning and Depression-Like Behaviour in Stressed Rats

**DOI:** 10.1371/journal.pone.0024263

**Published:** 2011-09-15

**Authors:** Suk-Yu Yau, Benson Wui-Man Lau, Jian-Bin Tong, Richard Wong, Yick-Pang Ching, Guang Qiu, Siu-Wa Tang, Tatia M. C. Lee, Kwok-Fai So

**Affiliations:** 1 Department of Anatomy, Li Ka Shing Faculty of Medicine, The University of Hong Kong, Hong Kong SAR, China; 2 State Key Laboratory of Brain and Cognitive Science, Li Ka Shing Faculty of Medicine, The University of Hong Kong, Hong Kong SAR, China; 3 Research Centre of Heart, Brain, Hormone and Health Aging, Li Ka Shing Faculty of Medicine, The University of Hong Kong, Hong Kong SAR, China; 4 Laboratory of Neuropsychology, The University of Hong Kong, Hong Kong SAR, China; 5 Laboratory of Cognitive Affective Neuroscience, The University of Hong Kong, Hong Kong SAR, China; 6 Joint Laboratory for Brain Function and Health (BFAH), Jinan University and The University of Hong Kong, Guanzhou, China; University of Queensland, Australia

## Abstract

Exercise promotes hippocampal neurogenesis and dendritic plasticity while stress shows the opposite effects, suggesting a possible mechanism for exercise to counteract stress. Changes in hippocampal neurogenesis and dendritic modification occur simultaneously in rats with stress or exercise; however, it is unclear whether neurogenesis or dendritic remodeling has a greater impact on mediating the effect of exercise on stress since they have been separately examined. Here we examined hippocampal cell proliferation in runners treated with different doses (low: 30 mg/kg; moderate: 40 mg/kg; high: 50 mg/kg) of corticosterone (CORT) for 14 days. Water maze task and forced swim tests were applied to assess hippocampal-dependent learning and depression-like behaviour respectively the day after the treatment. Repeated CORT treatment resulted in a graded increase in depression-like behaviour and impaired spatial learning that is associated with decreased hippocampal cell proliferation and BDNF levels. Running reversed these effects in rats treated with low or moderate, but not high doses of CORT. Using 40 mg/kg CORT-treated rats, we further studied the role of neurogenesis and dendritic remodeling in mediating the effects of exercise on stress. Co-labelling with BrdU (thymidine analog) /doublecortin (immature neuronal marker) showed that running increased neuronal differentiation in vehicle- and CORT-treated rats. Running also increased dendritic length and spine density in CA3 pyramidal neurons in 40 mg/kg CORT-treated rats. Ablation of neurogenesis with Ara-c infusion diminished the effect of running on restoring spatial learning and decreasing depression-like behaviour in 40 mg/kg CORT-treated animals in spite of dendritic and spine enhancement. but not normal runners with enhanced dendritic length. The results indicate that both restored hippocampal neurogenesis and dendritic remodelling within the hippocampus are essential for running to counteract stress.

## Introduction

The dentate region of the hippocampus is one of several neurogenic regions in the brain that continuously generate new neurons throughout adulthood. Functional integration of new neurons into existing circuits has been intensively investigated in recent years. Exercise and antidepressants can significantly increase hippocampal neurogenesis which is associated with improved cognitive function [1]. Neurotrophic factors and dendritic complexity of hippocampal neurons are also up-regulated by exercise [2], [3]. Conversely, chronic stress or exposure to exogenous glucocorticoid decreases hippocampal cell proliferation [4], induces dendritic atrophy [5], and impairs cognitive performance [6]. It is clear that many of the factors that are known to affect neurogenesis alter other aspects of brain structure, such as dendritic architecture and synapse number. Increase in both adult neurogenesis and dendritic remodeling, for example dendritic length and spine number, in mature neurons are likely to be involved in modulating hippocampal function. However, in previous studies, changes in neurogenesis and dendritic remodeling have only been examined separately.

It is known that elevated corticosteroids have profound effects on regulating the function and structure of the hippocampus. Detrimental effects of corticosteroids are proposed to be mediated by alterations in the expression of hippocampal brain-derived neurotrophic factor (BDNF) [7]. Accumulated plasma corticosterone (CORT) has also been reported to suppress neuronal differentiation of proliferating cells in the adult hippocampus [8]. In addition, stress-induced decreases in neurogenesis have been proposed to contribute to the pathophysiology of depressive disorders [9], [10]. There is evidence to demonstrate the important role of neurogenesis in regulating behaviours, such as spatial learning [11], depression-like behaviour [12] and even sexual behaviour [13], [14]. The association of improved spatial memory with increased hippocampal neurogenesis has been repeatedly demonstrated in exercised animals [1], [15]. These observations have suggested possible mechanism of how exercise counteracts the effects of stress. Nonetheless, in exercised rats, in addition to enhanced neurogenesis, exercise-induced dendritic remodeling may also contribute to behavioural responses to stress. Recent studies showed that ablation of hippocampal neurogenesis did not affect hippocampal-dependent spatial learning [16], [17], and it has been proposed that existing neurons or young neurons in granular cell layer may exert a compensatory role in maintaining hippocampal function in terms of hippocampal-dependent spatial learning and memory [17]. It is still unclear whether exercise-induced hippocampal neurogenesis or enhanced dendritic plasticity has a greater effect on maintaining hippocampal function under conditions of stress, or whether both factors contribute.

Animal models of stress, such as physical restraint [18], immobilization [19] and foot shock [6], are used for studying the effect of stress on hippocampal plasticity. However, individual variation in CORT levels between different animals exposed to the same stressor may increase experimental variability [20]. Repeated CORT injection in rodents has been suggested to be a reliable animal model for studying the role of stress in depressive disorders [21], [22]. In the present study, animals were treated with repeated injection of different doses (low, moderate and high) of CORT for fourteen days. We used this animal model to examine whether hippocampal neurogenesis, dendritic plasticity, or both, mediate the beneficial effects of exercise (i.e. wheel running) on stress.

Our results showed that exposure to CORT suppressed hippocampal neurogenesis and decreased spine density that was associated with spatial learning impairment. Running increased hippocampal neurogenesis, increased dendritic length, restored spine density and improved spatial learning in CORT-treated rats. Blockade of hippocampal neurogenesis abolished the beneficial effect of running in CORT-treated rats despite enhanced dendritic plasticity. The results suggest that new-born neurons and dendritic remodeling of established neurons both contribute to hippocampal plasticity and mediate the beneficial effects of exercise on stress.

## Results

### Running reversed CORT-suppressed hippocampal neurogenesis

**Figure 1:**
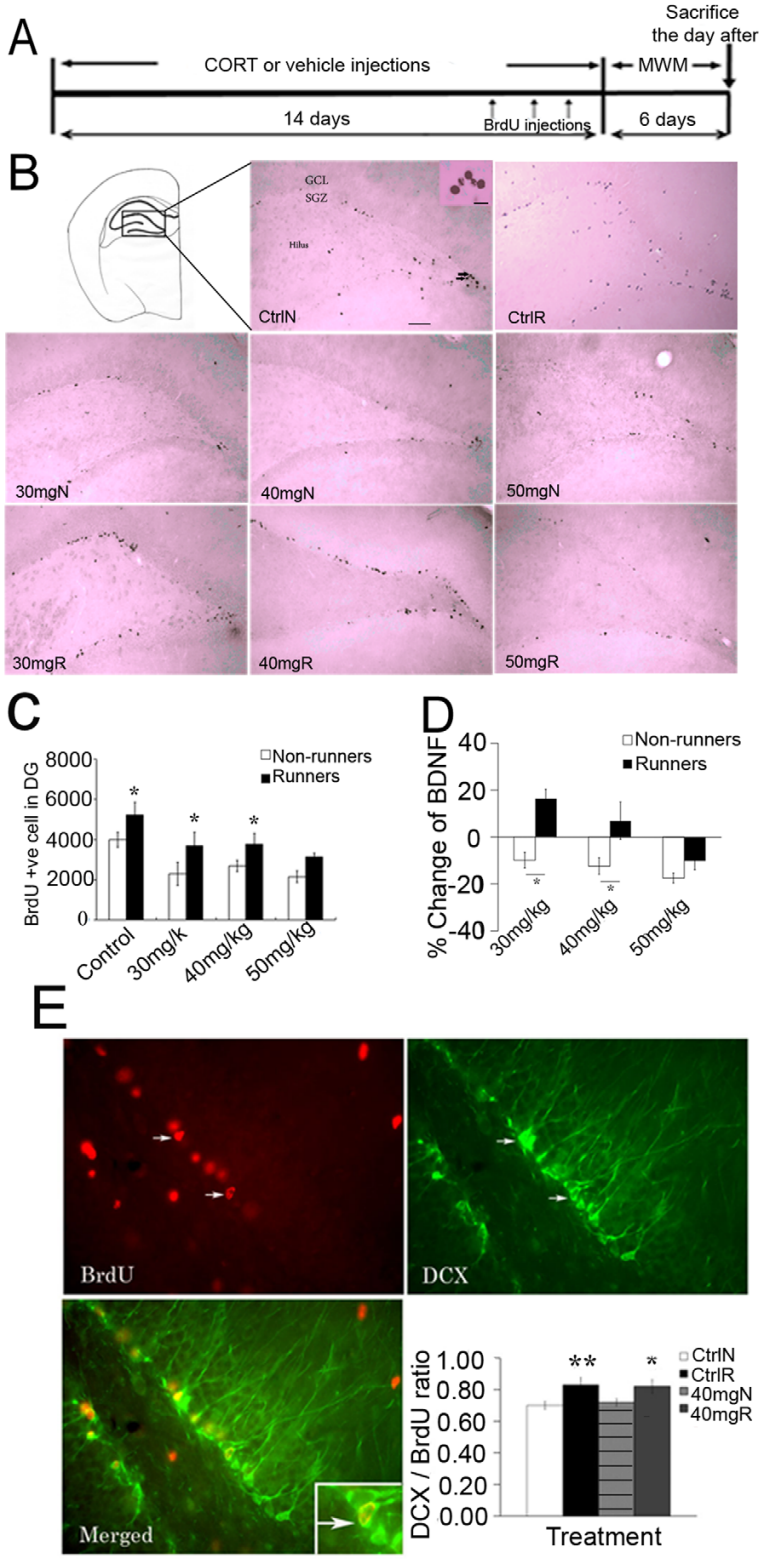
Hippocampal cell proliferation and neurogenesis in the dentate gyus. (**A**) A schematic diagram depicting experimental procedures for the treatment. MWM: Morris water maze (**B**) Representative images of BrdU-labelled cells. (**C**) CORT significantly suppressed hippocampal cell proliferation, whereas running reversed the decrease in vehicle-, 30 mg/kg and 40 mg/kg CORT-treated rats. *P<0.05 compared to non-runner counterparts. (**D**) There was no difference in hippocampal BDNF level between vehicle-treated runners and non-runners. However, BDNF level was decreased by CORT in a dose-dependent manner. Running up-regulated BDNF level in 30 mg/kg and 40 mg/kg CORT-treated rats, but not in 50 mg/kg CORT-treated rats. Data are expressed as percentage change±S.E.M. *P<0.005 compared to non-runner counterparts. (**E**) Running increased neuronal differentiation of newborn cells while CORT treatment showed no effect. *P<0.05, **P<0.005 compared to control non-runners. Values are respresented as mean±S.E.M. Ctrl: control; 30–50 mg: dosages of CORT treatment; N: non-runner; R:runner. Scale bar = 50 µm. N = 6–8/group.

Rats were treated with either vehicle or different doses of CORT respectively for 14 days and were injected with 50 mg/kg (body weight) of BrdU during the last three days of treatment. CORT treatment significantly reduced body weight gain and decreased adrenal gland weight indicating the efficacy of CORT injections (Fig. S1 and Supporting Information S1). Administration of CORT also significantly suppressed hippocampal neurogenesis while running significantly increased it (Figure 1C, effect of running, F_1,39_ = 21.861, P = 0.000543; effect of CORT, F_3,39_ = 9.1,P = 0.00018). Post hoc analysis revealed a significant decrease in BrdU-labeled cells in non-runners treated with 30, 40 and 50 mg/kg CORT compared to vehicle-treated non-runners respectively (P<0.05). Runners treated with vehicle, 30 mg/kg, 40 mg/kg CORT, but not runners treated with 50 mg/kg CORT showed a significant increase in BrdU-labeled cells in comparison to non-runner counterparts (P<0.05).

Running did not increase hippocampal BDNF level in vehicle-treated rats. However, CORT treatment decreased hippocampal BDNF levels in a dose-dependent manner (Figure 1D, effect of CORT, F_1,60_ = 8.191, P = 0.000142). Exercise up-regulated the BDNF level in 30 mg/kg and 40 mg/kg CORT-treated runners (effect of running, F_1,60_ = 29.544, P = 0.000141), but not in 50 mg/kg CORT-treated runners.

It is known that running increases cell proliferation, neuronal differentiation and survival of newly generated neurons in the hippocampus [1]. We examined neuronal differentiation 6 days after BrdU injection using doublecortin (DCX) staining in 40 mg/kg CORT-treated rats.

Running significantly increased the ratio of DCX to BrdU-labeled cells in vehicle-treated rats (effect of running, F_1,30_ = 13.447, P = 0.00111), while CORT treatment did not affect the ratio of DCX to BrdU-labeled cells (Figure 1E, effect of CORT, F_3,30_ = 0.03, P = 0.863). Runners treated with 40 mg/kg CORT showed a significant increase in DCX/BrdU ratio in comparison to vehicle-treated non-runners indicating increased production of new neurons after running. Owing to a decrease in cell proliferation in CORT-treated rats, the total number of newborn neurons in 40 mg/kg CORT-treated non-runners was decreased when compared with normal rats.

### CORT treatment increased depression-like behaviour in a dose-dependent manner

CORT treatment did not affect total running activity of the rats (**Fig S2**). CORT treatment also increased plasma CORT level in a dose dependent manner (Table 1, F_3,14_ = 3.078, P  = 0.016). The stress level of our CORT-treated rats was evaluated in comparison to chronic mild stress (Table S1 and Figure S3). To test whether different doses of CORT treatment would induce graded levels of depression-like behaviour in rats, a forced swim test was conducted the day after CORT treatment. The results indicated that CORT treatment increased depression-like behaviour (immobility time) while running decreased it.

**Table 1:** Plasma CORT level was increased in a dose-dependent manner after 7-day CORT treatment.

	Treatment			
	Vehicle	30 mg/kg	40 mg/kg	50 mg/kg
[CORT], ηg/ml	133±27	568±132*	860±225**	1488±444**

*P<0.05,

**P<0.005 compared to control. N = 4 group.

**Figure 2:**
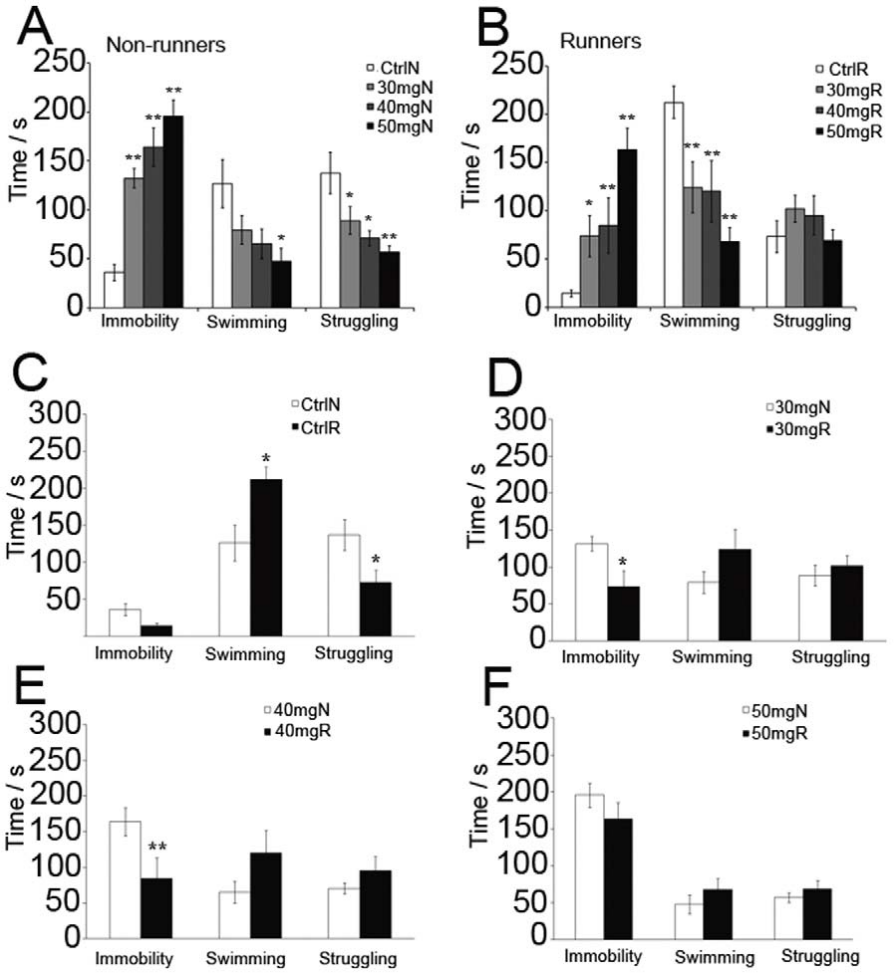
Effect of CORT injections on depression-like behavior. (**A** and **B**) Treatment with CORT increased immobility time in a dose-dependent manner while running decreased immobility time. * P<0.05; ** P<0.005 compared to control. (**C**) Running did not decrease immobility time but increased swimming time and decreased struggling time of vehicle-treated rats. (**D** and **E**) Post hoc analysis revealed that running significantly decreased immobility time in 30 mg/kg and 40 mg/kg CORT-treated rats when compared to non-runner counterparts. (**F**) Running showed no effect on immobility time in 50 mg/kg CORT-treated rats. *P<0.05, **P<0.005 compared to non-runner counterparts. N = 8–10/group for forced-swim test. Ctrl: control; 30–50 mg: dosages of CORT treatment; N: non-runners; R:runners.

Two-way ANOVA indicated a significant main effect of CORT on immobility time (Fig 2. effect of CORT, F_3,59_ = 22.007, P = 0.00279), swimming time (effect of CORT, F_3,59_ = 9.282, P = 0.000531) and struggling time (effect of CORT, F_3, 59_ = 2.96, P = 0.041). In addition, there was a significant main effect of running on immobility time (effect of running, F_1,59_ = 13.299, P = 0.00623) and swimming time (effect of running, F_1,59_ = 12.259, P = 0.000972). A significant interaction between running and CORT treatment was found in struggling time (running X CORT, F_3,59_ = 3.618, P = 0.019).

Post hoc analysis showed that CORT treatment in non-runners significantly increased immobility time in a dose-dependent manner indicating increased depression-like behaviour by CORT treatment (Fig 2A. P<0.005). In addition, CORT treatment in non-runners decreased swimming and struggling time when compared to vehicle-treated non-runners (P<0.05). Running in CORT-treated rats significantly increased immobility time and decreased swimming time when compared to runners with vehicle treatment (Fig 2B. P<0.05). CORT treatment did not affect struggling time in runners.

### Running reversed CORT-induced depression-like behaviour

In comparison between non-runners and runners, running did not decrease immobility time in vehicle-treated rats, but significantly increased swimming time and decreased struggling time (Fig 2C. P<0.05). However, running significantly decreased immobility time in rats treated with 30 mg/kg CORT and 40 mg/kg CORT (Fig 2D & E. P<0.05), but not 50 mg/kg CORT (Fig 2F. P>0.05). Running did not affect swimming and struggling time in CORT-treated rats when compared to non-runner counterparts. The data indicated that CORT treatment increased depression-like behaviour in forced swim test, while running was able to counteract depression-like behaviour induced by 30 mg/kg and 40 mg/kg CORT treatment.

### CORT treatment impaired spatial learning in non-runners

**Figure 3:**
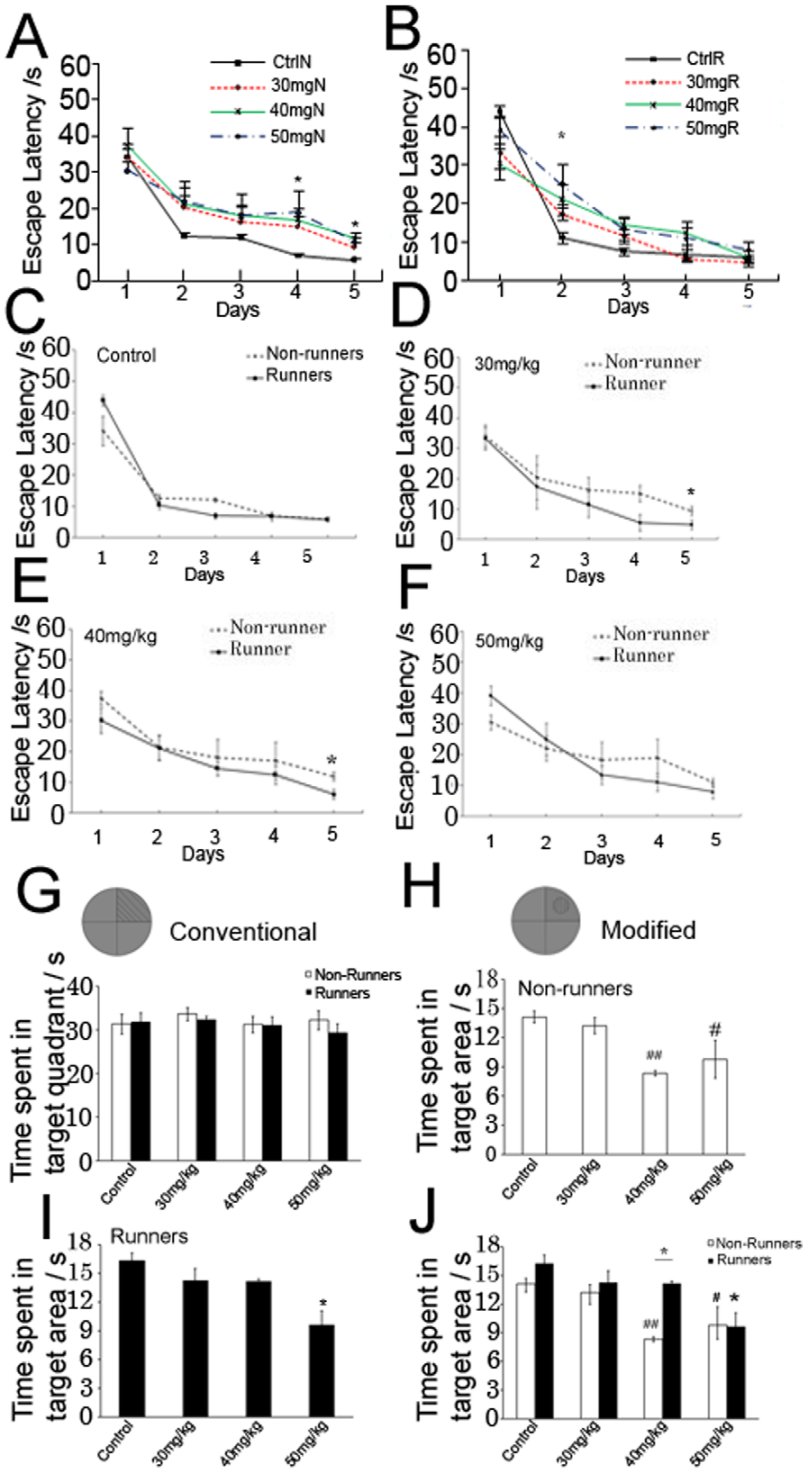
Performance in water maze task of CORT-treated non-runners and runners. (**A**) Non-runners treated with 30 mg/kg, 40 mg/kg and 50 mg/kg CORT showed impairment in spatial learning. (**B**) Runners with 50 mg/kg CORT treatment showed impairment in spatial learning compared to vehicle-treated runners. *P<0.05 compared to control. (**C**–**E**) Runners treated with 30 mg/kg and 40 mg/kg CORT showed significantly shorter escape latency compared to non-runner counterparts indicating improved spatial learning. (**F**) Running did not improve spatial learning in 50 mg/kg CORT-treated rats. *P<0.05 compared to non-runner conterparts. Data were analysed using conventional method in (G) and modified scoring method in (H–J). (**G**) There was no significant difference among groups using conventional method. (**H**) In the modified method, 40 mg/kg and 50 mg/kg CORT-treated non-runners showed a shorter time spent in target area compared with control non-runners. (**I**) Runners treated with 50 mg/kg CORT showed impairment in spatial learning compared to control runners. (**J**) This graph was a combination of (H) and (I) for representing the comparison between non-runners and runners. Running increased time spent in the target area in 40 mg/kg CORT-treated runners, but not in 50 mg/kg CORT-treated runners compared with non-runner counterparts. #P<0.05, ##P<0.005 compared to vehicle-treated non-runners. * P<0.05 compared to vehicle-treated runners. N = 8-10/group.

We then evaluated whether impairment in spatial memory was associated with a decrease in neurogenesis after CORT treatment. Rats were given water maze task for 5 days followed by a probe trial. Repeated measures ANOVA indicated that both runners and non-runners learned the position of hidden platform as indicated by a decreased escape latency across days (Figure 3, F_4,27_ = 47.631, P = 0.000553). There was a significant effect of CORT (effect of CORT: F_3,27_ = 3.489, P = 0.036) and effect of running on escape latency (effect of running: F_1,27_ = 7.279, P = 0.014). Post hoc analysis revealed that treatment with 30 mg/kg CORT in non-runners showed a significant increase in escape latency on day 5 (Fig 3A. P = 0.043) when compared to vehicle-treated non-runners. In addition, treatment with 40 mg/kg CORT and 50 mg/kg CORT in non-runners impaired spatial learning on day 4 (P = 0.047, P = 0.015) and day 5 (P = 0.002, P = 0.007) when compared to vehicle-treated non-runners. The data indicated that CORT treatment impaired spatial learning in non-runners.

### Running reversed impairment in spatial learning in 30 mg/kg and 40 mg/kg CORT-treated rats, but not 50 mg/kg CORT-treated rats

In contrast, runners treated with 30 mg/kg or 40 mg/kg CORT showed no difference in escape latency when compared to vehicle-treated runners while runners treated with 50 mg/kg CORT showed a significant longer escape latency on day 2 when compared with vehicle-treated runners indicating impaired spatial learning in 50 mg/kg CORT-treated runners (Figure 3B, P = 0.022).

In comparison between non-runners and runners, running did not decrease escape latency in vehicle-treated rats (Figure 3C, P>0.05). However, runners with 30 mg/kg or 40 mg/kg CORT treatment showed a significant decrease in escape latency on day 5 when compared to non-runner counterparts (Figure 3D & E, 30 mg/kg CORT: P = 0.026, 40 mg/kg CORT: P = 0.008 respectively). Runners with 50 mg/kg CORT treatment showed no difference in escape latency when compared to non-runner counterparts (Figure 3F). The data indicated that running was able to improve spatial learning in rats treated with 30 mg/kg or 40 mg/kg CORT, but not in the rats with 50 mg/kg CORT.

### CORT treatment impaired memory consolidation in 40 mg/kg and 50 mg/kg CORT-treated rats

To evaluate memory consolidation, a probe trial test was performed in the same maze without the platform. We observed no difference in memory retention among CORT-treated runners and non-runners using the conventional scoring method (Figure 3G). We then employed a modified scoring method, which used a circular area 20 cm in diameter measured from the centre of the platform. The main effect CORT and effect of running on time spent in target area was observed respectively (effect of CORT, F_3,39_ = 7.054, P = 0.000949; effect of running, F_1,39_ = 5.421, P = 0.0266). Post hoc tests showed that 40 mg/kg or 50 mg/kg CORT-treated non-runners decreased their preference for the target area (Figure 3H, P = 0.011, P = 0.00116 respectively) when compared with vehicle-treated rats. The data indicated that treatment with 40 mg/kg or 50 mg/kg CORT decreased preference for target area in non-runners.

### Running restored impairment in memory consolidation in 40 mg/kg CORT-treated rats

However, runners with 50 mg/kg CORT treatment showed significantly decreased time spent in the target area when compared with vehicle-treated runners (Figure 3I, P = 0.00179). In contrast, running increased time spent in the target area in 40 mg/kg CORT-treated rats (Figure 3J, effect of running, F_1,39_ = 5.421, P = 0.0266) The data indicated that running counteracted the detrimental effect of 40 mg/kg CORT treatment on memory consolidation (P = 0.00376). There was no difference in time spent in the target area between 50 mg/kg CORT-treated non-runners and runners (Figure 3 J, P = 0.943). Taken together, these results indicated that running decreased depression-like behaviour and improved spatial memory, which was associated with increased hippocampal neurogenesis in stressed rats as shown in Figure 1.

### Blocking hippocampal neurogenesis impaired spatial learning and increased depression-like behaviour in CORT-treated runners

**Figure 4:**
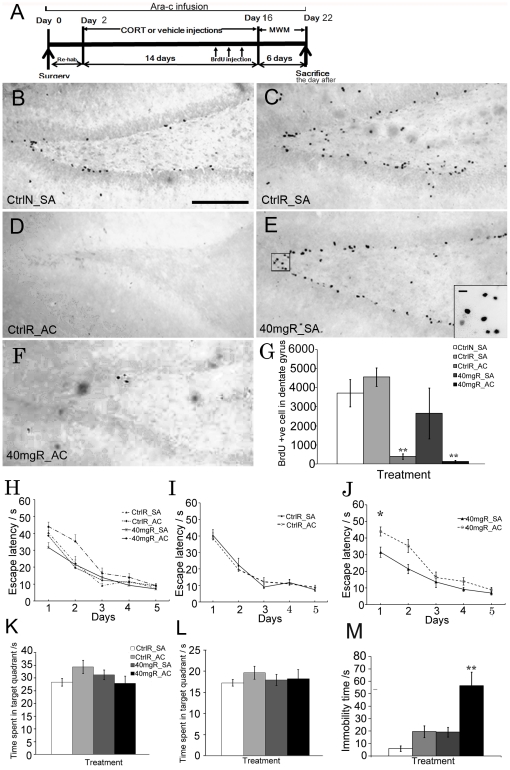
Effect of neurogenesis ablation on spatial memory and depression-like behavior. (**A**) A schematic diagram of intraventrucular infusion and treatment procedure; CORT treatment was started after two days of recovery from the surgical procedure. The behavioral test was then performed 24 hr after the treatment. MWM: Morris water maze (**B**–**F**) Representative images of BrdU staining. (**G**) Ara-c infusion significantly blocked hippocampal cell proliferation. (**H**) Spatial learning for different treatment groups. (**I**) Ara-c infusion did not affect spatial learning in vehicle-treated runners. (**J**) Blocking hippocampal neurogenesis in 40 mg/kg CORT-treated runners significantly impaired spatial learning compared to saline infused counterparts. Main effect of Ara-c infusion, * P<0.05 (**K** and **L**) Data were analyzed with the modified method. Memory consolidation was not altered by Ara-c infusion in either vehicle- or 40 mg/kg CORT-treated runners as indicated by analysis of conventional and modified method respectively in probe trial test. (**M**) Ara-c infusion significantly increased immobility time in 40 mg/kg CORT-treated runners indicating increased depression-like behavior. Main effect of Ara-c: **P<0.005. SA: Saline infusion; AC: Ara-c infusion. Scale bar  = 100 µm. N = 8–10/group.

To determine whether neurogenesis plays a role in the counteractive effect of running on stress, we blocked hippocampal neurogenesis with intraventricular infusion of an antimitotic agent: Ara-c (Figure 4A). Since we have shown that running was able to reverse the detrimental effects of 40 mg/kg CORT on hippocampal neurogenesis, hippocampal-dependent learning and memory and depression-like behaviour, animals with 40 mg/kg CORT treatment were used. Locomotor activity, which is not associated with hippocampal neurogenesis, was not affected by Ara-c infusion. The data indicated that Ara-c did not affect animal behaviour that was hippocampal neurogenesis-independent (Fig S4).

Figure 4B-G showed that Ara-c infusion robustly inhibited cell proliferation. All runners with Ara-c infusion showed a significant decrease in BrdU+ve cells when compared with the rats with saline infusion (Figure 4G, F_1,23_ = 50.826, P = 0.000888). All rats learned the position of the hidden platform (Figure 4H, F_4,100_ = 48.196, P = 0.000123). There was no significant difference in spatial learning between vehicle-treated non-runners and runners. This indicated that spatial learning in vehicle-treated runners was not affected by Ara-c (Figure 4I). However, spatial learning was impaired by Ara-c infusion in 40 mg/kg CORT-treated runners when compared with saline-infused counterpart as indicated by significant increase in escape latency in Ara-c infused runners with CORT treatment when compared to saline-infused counterpart (Figure 4J, effect of Ara-c, F_1,100_ = 5.528, P = 0.032, CORT X Ara-c infusion, F_1,100_ = 4.61, P = 0.047).

Figure 4K and 4L showed no alteration in the probe trial with Ara-c infusion using the modified scoring method. Blocking neurogenesis significantly increased immobility time in 40 mg/kg CORT-treated rats with Ara-c infusion when compared with vehicle-treated non-runners with saline infusion (Figure 4M, effect of Ara-c, F_1,15_ = 13.406, P = 0.00326; effect of CORT, F_1,15_ = 12.963, P = 0.00364). The data indicated that Ara-c diminished the beneficial effect of running on reducing depression-like behaviour in 40 mg/kg CORT-treated runners.

### Running increased dendritic length and spine density in 40 mg/kg CORT-treated rats

Our results demonstrated that running reduced depression-like behaviour and improved spatial memory and that both behaviours required hippocampal neurogenesis in CORT-treated rats, but not in normal rats. We therefore chose 40 mg/kg CORT treatment for further experiments to examine 1) whether running could induce dendritic remodeling in the hippocampus in 40 mg/kg CORT-treated rats; and 2) explored whether there would be structural changes in runners infused with Ara-c.

**Figure 5:**
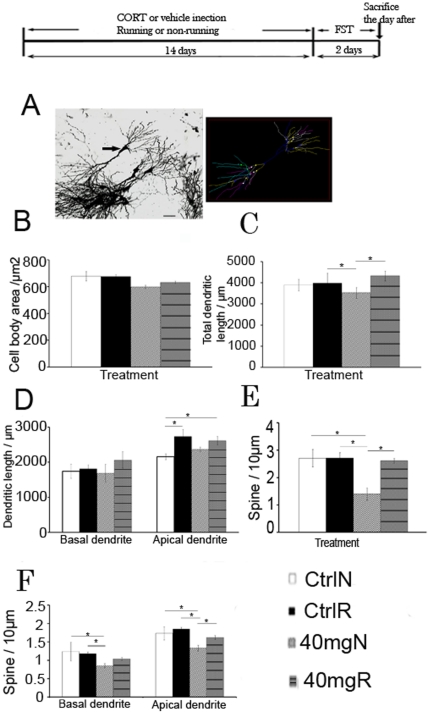
Dendritic remodeling of hippocampal CA3 pyramidal neurons. (**A**) Left: A representative image of a selected neuron for analysis; Right: A neuronal morphology by using Neurolucida software (**B**) The soma size was not affacted by running and CORT treatment. (**C**) CORT treatment significantly decreased total dendritic length while running reversed the decrease. (**D**) The basal dendritic length was not affected by running and CORT treatmnet. The apical dendritic length was significantly increased by running in vehicle or CORT treated rats. (**E**) Running did not increase spine density in vehicle-treated rats, but restored the decrease in 40 mg/kg CORT-treated rats. **(F**) Treatment with 40 mg/kg CORT significantly decreased spine density in basal dendrites and apical dendrites. Running restored the decrease in spine density in 40 mg/kg CORT-treated rats. *P<0.05; **P<0.005. N = 3 for control non-runnes, N = 5-8 for the rest groups. Ctrl: control; 40 mg: 40 mg/kg CORT; N: non-runner; R:runner. Scale bar = 100 µm. FST: forced swim test.

We found that running and CORT treatment did not affect cell body area or dendritic length in the granular cell layer; however, spine density was significantly increased in the outer granular cell layer of CORT-treated runners (data not shown). Modification of dendrites can modulate the integration of each individual neuron's synaptic input, which may in turn alter hippocampal plasticity. Chronic stress and CORT-exposure for 21 days produced dendritic atrophy in hippocampal neurons of the CA3 region [5]. In light of the specificity of CORT on CA3 pyramidal neurons as reported from the literature [23], we analyzed Golgi-impregnated CA3 pyramidal cells (Figure 5A). Analysis showed that neither running nor CORT treatment affected cell body area (Figure 5B, effect of running, F_1,26_ = 0.365, P = 0.552; effect of CORT, F_1,26_ = 1.404, P = 0.249). There was a main effect of running on increasing total dendritic length, but no effect of CORT was observed (Figure 5C, effect of running, F_1,20_ = 10.386, P = 0.00532; effect of CORT, F_1,20_ = 0.887, P = 0.360). There was no difference in total dendritic length between 40 mg/kg CORT non-runners and vehicle-treated non-runners; however, 40 mg/kg CORT-treated non-runners showed a significant decrease in total dendritic length when compared with vehicle-treated runners (F_3,19_ = 5.016; P = 0.009). Runners with 40 mg/kg CORT treatment showed a significant increase in total dendritic length when compared with non-runner counterparts (F_3,19_ = 5.016; P = 0.002). Running did not affect dendritic length of the basal dendrite, but significantly increased apical dendritic length in vehicle-treated and CORT-treated runners (Figure 5D, effect of running, F_1,22_ = 7.915, P = 0.00115).

Treatment with 40 mg/kg CORT decreased spine density in non-runners (Figure 5E, effect of CORT, F_1,19_ = 10.792, P = 0.00501) while running restored the decrease in spine density in 40 mg/kg CORT-treated rats (effect of running, F_1,19_ = 8.011, P = 0.0127; running X CORT, F_1,19_ = 1.625, P = 0.0132). Running did not increase total spine density in vehicle-treated rats (Figure 5E). Spine density in basal and apical dendrites was also not affected by running in vehicle-treated rats (Figure 5F), but spine density was significantly decreased by 40 mg/kg CORT treatment compared with vehicle-treated rats (Figure 5F, basal dendrites: F_1,19_ = 5.567, P = 0.0313, apical dendrite F_1,19_ = 11.031, P = 0.00465). The 40 mg/kg CORT-treated non-runners showed a significant decrease in basal and apical spine density when compared with vehicle-treated runners and non-runners respectively (Figure 5F, P<0.05). Running reversed the decrease in spine density in 40 mg/kg CORT-treated rats (effect of running, F_1,19_ = 4.667, P = 0.0473).

### Ara-c infusion did not cause dendritic remodeling in the pyramidal neurons and did not affect the effect of running on dendritic remodeling

**Figure 6:**
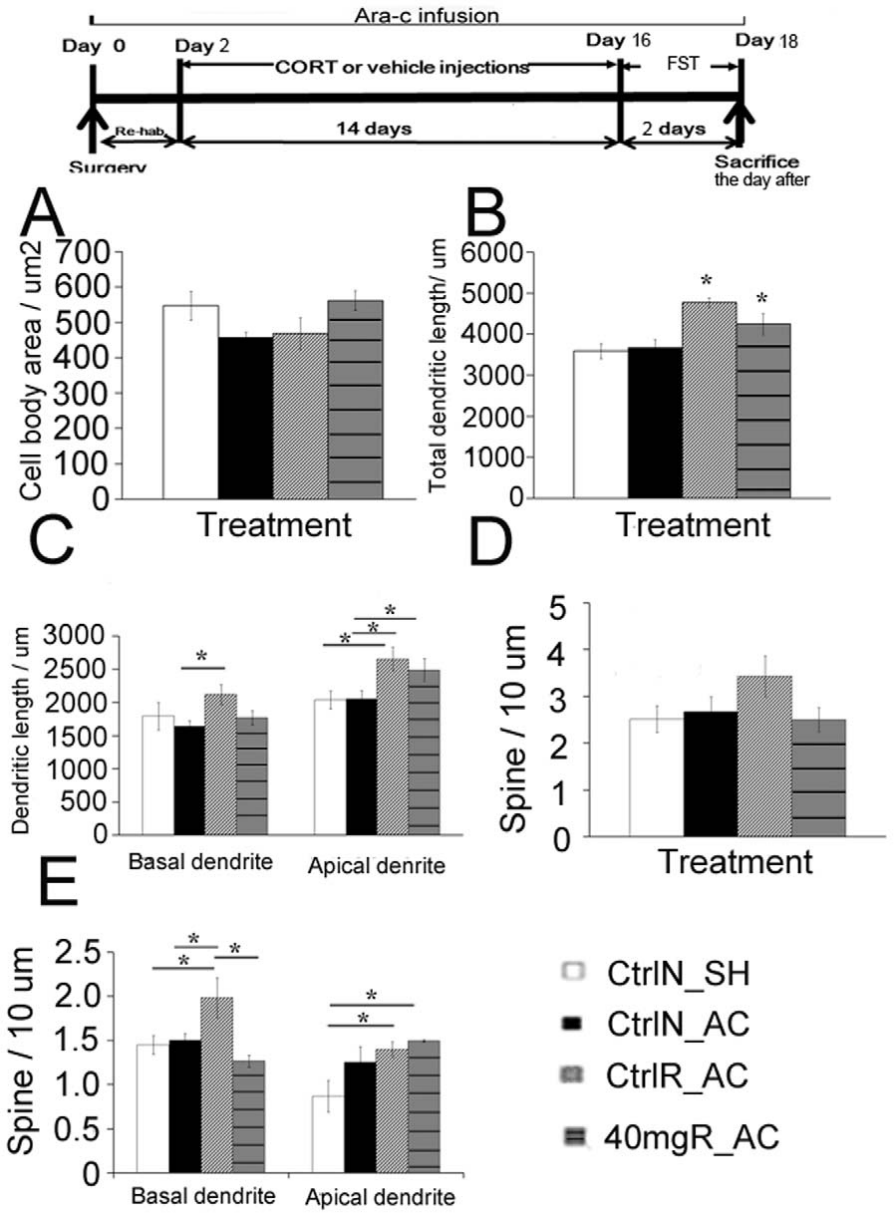
Structural remodeling of CA3 pyramidal neurons in Ara-c-infused runners and non-runners. (**A**) There was no significant difference in the soma size among groups. (**B**) The total dendrite length was not affected by Ara-c infusion in vehicle-treated non-runners. A significant increase in dendritic length was observed in both vehicle-treated runners with Ara-c infusion and 40 mg/kg CORT-treated runners with Ara-c infusion when compared with vehicle-treated non-runners with sham-operation or vehicle-treated non-runners with Ara-c infusion. (**C**) Dendritic length of basal and apical dendrite was increased in vehicle-treated runners with Ara-c infusion when compared to non-runners. (**D**) There was no change in spine density in Ara-c infused non-runners with vehicle treatment. Running increased spine density in vehicle-treated and 40 mg/kg CORT-treated rats. (**E**) Vehicle-treated runners with Ara-c infusion showed a significant increase in spine density in basal dendrite when compared with other groups respectively. Ara-c infused runners treated with either vehicle or 40 mg/kg CORT showed a significant increase in spine density in apical dendrite when compared with vehicle-treated non-runners with sham operation. Values are represented as mean±S.E.M. *P<0.05. N = 5-6/group for CtrlN_SH, CtrlN_AC and CtrlR_AC group. N = 3/group for 40 mgR_AC group. CtrlN_SH: Vehicle-treated non-runners with sham-operation. CtrlN_AC: Vehicle-treated non-runners with Ara-c infusion; CtrlR_AC: Vehicle-treated runners with Ara-c infusion: 40 mgR_AC: 40 mg/kg CORT-treated runners with Ara-c infusion. FST: forced swim test.

Based on the previous results, we speculated that the effect of running on stress required both dendritic remodeling and a normal level of hippocampal neurogenesis. Golgi analysis in Ara-c infused rats was therefore conducted to 1) further support the hypothesis and 2) confirm that Ara-c infusion did not affect dendritic plasticity. Results showed that there was no significant difference among groups in cell body area (Fig 6A). Ara-c infusion did not affect dendritic length or spine density (Student t-test, P>0.05 in comparison between control non-runners with shame operation and control non-runners with Ara-c infusion). After blockade of hippocampal neurogenesis by Ara-c infusion, running still exerted its positive influence on dendritic length in runners. After Ara-c infusion, significant differences in total dendritic length and apical dendritic length were observed in vehicle-treated or 40 mg/kg CORT-treated runners when compared with vehicle-treated non-runners (Figures 6B & C, Total: F_3,17_ = 8.867, P = 0.00184; Apical: F_3,17_ = 4.646, P = 0.0187; Basal: F_3,17_ = 1.721, P = 0.208). The vehicle-treated runners with Ara-c infusion and 40 mg/kg CORT-treated runners with Ara-c infusion showed a significant increase in total dendritic length when compared vehicle-treated non-runners with either sham operation or Ara-c infusion respectively (Figure 6B, P<0.05). Apical dendritic lengths were significantly increased in vehicle-treated and 40 mg/kg CORT-treated runners after Ara-c infusion when compared with vehicle-treated non-runners with either sham operation or Ara-c infusion (Figure 6C, P<0.05). There was no significant difference in total spine density among groups (Figure 6D, F_3,17_ = 1.500, P = 0.258). However, spine density in basal dendrites was significantly increased by running in vehicle-treated runners with Ara-c infusion when compared to the other three groups respectively (Figure 6E, Basal: F_3,17_ = 4.489, P = 0.00305). In apical dendrites, there were significant differences in spine density in vehicle-treated and 40 mg/kg CORT-treated runners with Ara-c infusion when compared with vehicle-treated non-runners with sham-operation (P = 0.027). These data indicated that running was able to increase dendrite length and spine density in vehicle- and CORT-treated rats in the absence of neurogenesis.

Our results indicated that decreased dendritic remodeling and suppressed neurogenesis was associated with behavioural deficits in CORT-treated rats. Treatment with Ara-c did not affect dendritic length or spine density in vehicle-treated rats. However, the effect of running on dendritic remodeling still occurred in both vehicle-treated and 40 mg/kg CORT-treated runners with Ara-c infusion.

## Discussion

Using a reliable stress model induced by repeated CORT injection [21], [22], we investigated the effects of stress on depression, learning and memory. We also determined whether the beneficial effect of running on stress was mediated by restored hippocampal neurogenesis and induced dendritic remodeling. Our results showed that running restored spatial learning to normal levels and decreased depression-like behaviour in CORT-treated rats. The effects of running were associated with restored hippocampal neurogenesis, increased BDNF levels and enhanced dendritic plasticity. Blockade of neurogenesis diminished behavioural improvements in spatial learning and depression-like behaviour in 40 mg/kg CORT-treated runners which showed increased dendritic length and spine density, but not in normal runners showing enhanced dendritic length in the CA3 pyramidal neurons The findings suggest that enhanced dendritic plasticity and restored hippocampal neurogenesis, which represents structural plasticity of the hippocampus (an important requirement for maintaining hippocampal function), may enable exercised animals to be better adjusted to stress.

### Increased hippocampal neurogenesis was required for improving depression-like behaviour and spatial memory in stressed animals

The efficacy of exercise for treating stress-related illnesses, especially depression, is well documented [24]. It has been hypothesized that depressive disorders might be linked to decreased hippocampal neurogenesis [6], [25]. We demonstrated that normalized hippocampal neurogenesis was associated with improvements in spatial memory and depression-like behaviour in 2-week CORT-treated runners. Other groups found that the beneficial effects of exercise on depression-like behaviour is linked to increased hippocampal neurogenesis in genetically depressed rats and depressed-animal models [26], [27]. Disrupting hippocampal neurogenesis with x-irradiation blocked the effect of antidepressant treatment on depression-like phenotypes [12], [28]. Others reported that two weeks of either irradiation or injection of methylazoxymethanol caused memory impairment and decreased hippocampus neurogenesis [29], [30]. Infusion of Ara-c for two weeks, in fact, blocked not only proliferating cells, but also ongoing neurogenesis (immature neurons during treatment period). Our results showed that, after blocking hippocampal neurogenesis, the behavioural benefits of running disappeared in CORT-treated runners. These data reinforced the hypothesis that increased neurogenesis may play a role in improving hippocampal-related behaviours under stress conditions.

There are studies showing that BDNF plays a critical role in not only synaptic plasticity, but also in neuronal differentiation and survival [31]–[33]. In phenotypic analysis of newborn cells using doublecortin and BrdU co-labelling, our results showed that running significantly increased neuronal production. Although 14-day treatment may not be long enough for newborn cells to be fully differentiated into mature neurons, the function of immature neurons should not be neglected. Neuronal maturation takes ∼3–4 wk with new neurons being functionally integrated into existing circuits [34]. However, it has been reported that immature neurons could be involved in hippocampal information processing before they differentiate [35]. Immature neurons in the hippocampus display a greater propensity for synaptic plasticity compared to old neurons having enhanced excitability and a lower threshold for LTP induction [36], [37]. Immature neurons may also contribute to hippocampal function by secreting neurotrophic factors for mature neurons to establish their synaptic connection. This is supported by findings that neural stem cells not only differentiate into neuron-like cells, but also produce various trophic factors which support other cell types [38], [39].

**Figure 7:**
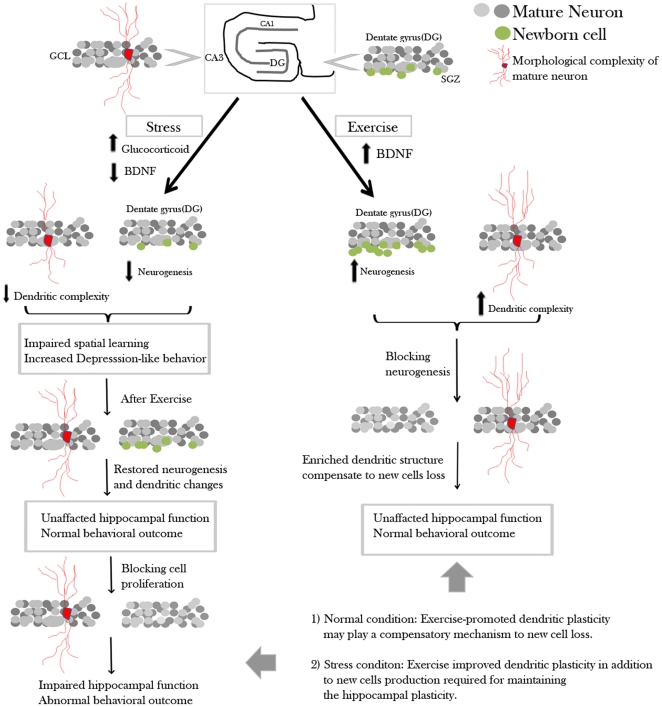
Schematic diagram showing the hypothesis of the counteracting mechanism of running on stress. Exposure to stress increases glucocorticoid level and decreases BDNF, which results in decreased hippocampal neurogenesis and dendritic retraction. In normal rats, existing neurons may be able to maintain hippocampal plasticity. In stressed rats, intact hippocampal plasticity, which maintained by continuous production of new neurons and dendritic remodeling, may mediate the beneficial effects of running on stress.

Numerous studies indicate that spatial learning and neurogenesis are related. New proliferating cells are involved in acquiring new information and a higher baseline level of neurogenesis correlates with better performance in learning phase, but does not affect probe trial performance [40]. It has been suggested that a cognitive deficit appears when the number of new cells in the hippocampus decreases to a critical level [12]. However, in our data, blockade of hippocampal neurogenesis diminished the positive effect of running in CORT-treated runners but not in normal runners. In other studies, impaired spatial learning was also not observed in animals whose neurogenesis had been reduced by treatment with anti-mitotic drugs or irradiation [16], [41]. Wojtowicz *et al*. [17] found that irradiation-induced reduction in hippocampal neurogenesis in exercised rats did not impair spatial learning. They hypothesized that mature neurons and remaining young neurons partially compensated for the loss of new cell production within the hippocampus. Our result demonstrated an enhanced apical dendritic length and increased spine density of CA3 pyramidal neurons in vehicle-treated runners with Ara-c infusion. However, this is not observed in the dentate region (data not shown). Our data support Woitowicz *et al*. 's suggestions that some form of compensation could occur in the case of neuronal depletion. Our data may suggest that, in normal conditions, dendritic remodeling of pyramidal neurons in the CA3 region may compensate for the loss of new cells in the dentate gyrus to maintain hippocampal plasticity, thus preventing behavioural deficits in normal runners (Figure 7).

### Neurogenesis and dendritic remodeling was required for exercise to counteract stress

Structural plasticity of the hippocampus involves reorganization of synapses and changes in dendritic arborisation in the CA3 and CA1 areas [42], while cellular plasticity involves neurogenesis in the dentate gyrus. Exercise is shown to up-regulate the expression of BDNF, IGF-1 and VEGF [43], [44] and enhance dendritic length and numbers of spines in the hippocampus [45]. Stress-induced reduction of neurotrophic factors such as BDNF may alter dendritic structure and decrease neurogenesis [46]. This may in turn affect synaptic function in the hippocampus [47]. Therefore, it is generally assumed that, in addition to dendritic rearrangement of mature neurons, hippocampal neurogenesis may also be involved in modulating existing hippocampal neuronal circuits [48].

Recently, it has been found that administration of chronic fluoxetine not only increases cell proliferation, but also accelerates maturation of newborn neurons which display more complex dendritic arborisation [49]. It is possible that running also accelerates maturation of newborn neurons in our CORT-treated rats during the treatment period since it has been reported that in a mouse model with genetically down-regulated hippocampal cell proliferation, exercise reversed the decrease in hippocampal proliferating cells and enhanced dendritic arborisation of newly generated neurons[50]. Although we did not examine the dendritic arborisation of newly generated neurons in the GCL, our data demonstrated an association between improved hippocampal function (improved spatial learning and decreased depression-like behaviour) and structural changes in the hippocampus (restored hippocampal neurogenesis in the dentate region and reversed dendritic atrophy in the CA3 region) in CORT-treated runners. Also, wheel running induced dendritic remodeling in the CA3 region in CORT-treated rats with or without neurogenesis ablation. However, neurogenesis ablation diminished the effect of exercise on behavioural improvements in terms of spatial learning and depression-like behaviour in CORT-treated, but not normal, rats. The result indicated that beneficial effects of exercise on stress involved both restored hippocampal neurogenesis and dendritic remodeling.

It has been reported in a rat model using 2-week exposure to unpredictable chronic stress that antidepressants improved depressive behaviours that is neurogenesis independent, but is associated with dendritic remodeling in the prefrontal cortex and hippocampus [51]. It is possible that exercise and antidepressants exert their therapeutic effects via different mechanisms. Although we did not examine structural changes in the prefrontal cortex, present results may support others' view that behavioural deficits in depressed subjects may result from dysfunction of hippocampal plasticity (e.g. decreased hippocampal neurogenesis and dendritic atrophy). This dysfunction may in turn disrupt neuronal communication with other brain regions, such as the prefrontal cortex and amygdala [51], [52] since these brain regions are interconnected and influence each other [53].

To the best of our knowledge, we are the first group to show dose-dependent effects of CORT treatment on hippocampal neurogenesis. In fact, we have also demonstrated an inverted U-dose response of hippocampal proliferating cells to CORT treatment ranging from 5 mg/kg to 40 mg/kg (Yau et al., To be submitted). The mechanism whereby CORT affects hippocampal progenitor cells remains poorly understood. Absence of mineralcorticoid receptors and glucocorticoid receptors on granular precursor cells [54] may imply that glucocorticoid regulates the proliferative process indirectly. Stimulated hippocampal glutamate release in adulthood and activation of NMDA receptors by elevated glucocorticoids is suggested to inhibit cell proliferation in the dentate gyrus [55], [56]. In this study, we only showed that running increased dendritic length and spine density in 40 mg/kg CORT-treated rats. From a later study in our group, we observed that running was unable to reverse the decrease in spine density in 50 mg/kg CORT-treated rats (CD Lee, 2010, Master Dissertation). Taken together, our results suggest that basal levels of new cells in the dentate gyrus, together with dendritic remodeling of mature neurons, may be required to maintain neuronal plasticity in the hippocampus, and therefore contributes to behavioural improvement in CORT-treated rats after running (Figure 7).

In summary, our data indicate that, in a CORT-induced animal model of stress, exercise promoted hippocampal neurogenesis and triggered dendritic remodeling that were both critical for the beneficial effect of exercise on stress. Our work may suggest a plasticity mechanism underlying the beneficial effect of physical exercise on stress which may involve not only restored hippocampal neurogenesis, but also dendritic remodeling of existing neurons within the hippocampus.

## Materials and Methods

### Animals and housing conditions

All experimental procedures were approved by the Committee on the Use of Live Animals in Teaching and Research (CULATR 1449-07, 1575-07,), The University of Hong Kong. Adult male Sprague-Dawley rats (250±20 g) were housed individually with access to a running wheel or a locked wheel. All rats were kept on a 12 hr light-dark cycle with *ad libitum* access to food and water. Rats were treated with sesame oil or different doses of CORT for 14 days. Bromodeoxyuridine (BrdU, 50 mg/kg body weight) was dissolved in normal saline and administered intraperitoneally during the last 3 days of treatment. This was followed by water maze task or forced-swim test. Basically, the animals were divided into groups as: Non-runners + vehicle/CORT and Runners + Vehicle/CORT. Corticosterone (Sigma, St Louis) was prepared daily and injected subcutaneously according to the method of Hellsten *et al*.[57].

### Exercise training

Runners were housed singly in cages equipped with running wheels (diameter, 31.8 cm; width, 10 cm; Nalgene Nunc International, NY). Rats were allowed to run during the treatment period and wheels were locked during behavioural testing. Wheel revolutions were recorded using VitalViewer software (Mini Mitter Company, Inc, OR). Non-runners were housed in the identical cages with a locked wheel.

### Water maze task

The Morris water maze task was used for assessing hippocampal-dependent learning and memory of the rats. Water was made opaque by adding nontoxic ink. Animals were brought into the room 30 min prior to the testing and received four trials per day. Latency was recorded when rats reached the platform. If the rat did not reach the platform within 60 s, it was assigned a score of 60 s. After remaining on the platform for 20 s, the animal was dried and returned to its cage and was given a 4-min inter-trial rest. A probe trial was conducted on day 6. For the conventional scoring method in the probe trial, the circular tank was divided into four equal quadrants; in the e modified scoring method, the target circle was defined as an area with 20 cm diameter as measured from the centre of the platform. Data were analyzed by a trained observer who was blinded to experimental conditions.

### Forced swim test

This test is widely used for assessing the learned helplessness behaviour as a valid indicator for depression. The test was conducted according to the method of Porsolt *et al*. [58]. Tests were performed the day after treatment. Rats were placed in a cylinder (60cm height X 25 cm diameter) for 15 min on day 1 and 5 min on day 2. Day 2 session was video-taped for scoring by an observer blind to the treatment conditions.

### Tissue preparation and immunohistochemistry

Animals were anaesthetized using intra-peritoneal injection of overdose of pentobarbitone and perfused transcardially with normal saline, followed by 4% paraformaldehyde. The brain was post-fixed at 4°C overnight and was left in 30% sucrose until it sank. Coronal sections of 40 µm thickness were cut through the hippocampus from the bregma −3.30 mm to −4.52 mm [59]. BrdU staining was performed as described in our early study by Lau *et al.* [60]. In brief, sections were placed in 0.01 M citric acid (pH = 6.0) at 95°C for 30 min in a water bath. After three 0.01 M PBS rinses, sections were incubated in 1 M HCl for 30 min at 37°C followed by boric acid buffer (pH = 8.5, 15 min) at room temperature. After three 0.01 M PBS rinses, sections were incubated in blocking solutions for 1 hour and incubated with mouse anti-BrdU antibody (1:1000, Roche Molecular Biochemicals, Essex, UK) overnight at room temperature. Sections were washed and incubated with biotinylated goat anti-mouse antibody (1:200, Vector Laboratories, Inc Burlingame, CA). Finally, the sections were incubated with an avidin-biotin complex (Vector Laboratories) and BrdU immunoreactivity was visualized with diaminobenzidine. Brain sections were counted in a one-in-twelve series of coronal sections as described by Gould *et al*. [61]. For immunofluorescence staining, sections were incubated with primary antibodies: rat anti-BrdU antibody (1∶1000, Abcam, USA) and rabbit anti-doublecortin (1∶100, Abcam, USA). Section were rinsed and then incubated with Alexa 488-conjugated goat anti-rabbit and 568-conjugated goat anti-rat (1∶200 Molecular Probes, Eugene, OR) overnight at room temperature. After rinsing, sections were coversliped.

### Data quantification of cell proliferation and neurogenesis

Coronal brain sections containing BrdU-positive cells were counted through a 40X objective using the StereoInvestigator (MicroBrightField, Williston,VT). The number of positive cells was counted within the granular cell layer (GCL) and two cell diameters below the GCL, ignoring the cells in the uppermost focal plane. The resulting numbers were then multiplied by 12 to obtain the estimated total number of BrdU-positive cells per GCL. BrdU-labeled cell counts were performed by a colleague in a sample-blinded manner. For the phenotype analysis, 30 BrdU-positive cells per animal randomly was selected and analyzed for the co-expression of BrdU and doublecortin. Ratios of DCX to BrdU labelled cells were determined in a blinded manner.

### Intraventricular infusion

Rats were infused using the protocol of Jung *et al*. [62]. Rats were anaesthetized by intraperitoneal injection of a mixture of ketamine/xylazine (85/5 mg/kg). The anaesthetized rats were infused with either 2% cytosine-b-D-arabinofuranoside (Ara-C): an antimitotic agent or vehicle (0.9% saline), using an osmotic minipump (Alzet, model 2004; Palo Alto, CA; flow rate 0.25 µl/h for 28 days) throughout the experimental period. A cannula was implanted into the left lateral ventricle (AP: 2.0 mm L: 1.5 mm, DV: 3.5 mm). CORT treatment and running were started after two recovery days from the surgical procedures.

### Golgi staining and analysis

Golgi staining was performed with the FD Rapid Golgistain™ Kit (FD Neurotechnologies, MD) according to the manufacturer's instructions. In brief, freshly dissected brain was kept in impregnation solution A and B containing potassium dichromate and chromate for two weeks at room temperature. After being replaced with solution C for 48 hours at 4°C, brain tissues were sectioned into 150 µm and fixed to gelatine coated slides. The mixture of solution D, E and distilled water (1∶1∶2 ratio) were used for staining pyramidal neurons for 10 mins, followed by hydration in 50%, 75%, 95% ethanol and absolute ethanol. The brain sections were finally cleared in xylene and covered by coverslip in permount. Five neurons from 150 um-thick sections were analyzed using Neurolucida (MicroBrightField, USA) and selected according to the method described by Woolley et al. [23]. Only neurons located in the CA3 region of the dorsal hippocampus were selected for analysis. The selected neurons have to be relatively isolated from neighbouring impregnated neurons to avoid interference with analysis. Cells bodies should be located in the middle part of the section thickness so as to minimize the cut of branch segments. Also, the neurons should be consistently and darkly impregnated along the entire extent of all dendrites. The spine density was estimated by randomly selecting high-magnification tracing of a >10 µm-long terminal segment of the basal/apical dendritic branch. Three to five tertiary apical dendrites and basal dendrites with at least one branch point were selected for counting. The visible spines along the branch segment were counted and data were expressed as number/10 µm.

### The expression of BDNF level in the hippocampus

Protein expression levels of hippocampal BDNF were measured using commercial ELISA kit (Millipore Corporation, USA). The procedure was performed according to the manufacturer's instructions. In brief, 50 µg protein of each sample and standard was loaded into microtiter plates. Plates were covered by plate sealer and incubated at 4°C overnight. Plates were then washed four times with wash buffer. Diluted biotinylated mouse anti-BDNF monoclonal antibody (1:1000 diluted with diluents) was added into each well and incubated at room temperature for 3 hr on shaker. After wash, diluted streptavidin-HRP conjugate solution was added and incubated at room temperature for 1 hour. After adding substrate and stop solution, the optical density was determined (absorbance at 450 nm) by a plate reader. The standard curve showed direct relationship between optical density and BDNF concentration. To eliminate the inter-assay variation, the BDNF level was presented as percentage of control.

### Plasma CORT measurement

Blood samples were collected 1-hr after injection of CORT at day 7. Animals were anaesthetized using the mixture of katemine and xylazine (2∶1 ratio) and blood was collected from the tail vein within 3 min using a heparinized needle. Samples were centrifuged (2000 rpm for 20 min at 4°C). Plasma aliquots were stored at -80°C until use. The CORT level was determined using a CorrelateEIA corticosterone kit (Assay design, USA). Measurements were performed according to the manufacturer's instruction. Briefly, plasma samples were diluted 1∶500 with asssay buffer. 100 µl sample and standard were added into the appropriate well, followed by corticosterone-conjugate and corticosterone antibody. Plates were incubated at room temperature on shaker for 2 hr at 500 rpm. After 4 washes of the wells, substrate and stop solution was added. Corticosterone concentration was measure at 405 nm and 570 nm.

### Statistical analysis

A two-way ANOVA with exercise and corticosterone as factors was performed with LSD post-hoc test for analysing the quantification of BrdU-, DCX-positive cells and the Golgi-stained cells in rats treated with either vehicle or corticosterone. One-way ANOVA was used for analysing data from the plasma corticosterone test and Golgi analysis in rats with Ara-c infusion. The water maze acquisition data were analyzed using repeated measures ANOVA with day as within-factor and exercise, corticosterone treatment as between-subjects factor. For the intraventricular infusion part, water maze data were analyzed using repeated measure ANOVA with corticosterone treatment and Ara-c infusion as between-subjects factor. Two group comparison was performed using Student's t-test.

## Supporting Information

Figure S1 **Body weight and adrenal weight change of the rats.** a) Non-runners with CORT treatment showed significant decrease in body weight compared to vehicle-treated non-runners. b) Runners with CORT treatments also showed robust decrease in body weight again during the treatment period. C) Adrenal to body weight ratio was significantly decreased by CORT treatment. Values are represented as mean±S.E.M. * p<0.005 compared to the vehicle-treated rats. N = 6–10/group.
(TIF)

Figure S2 **Running activity of the runners.** There was no significant difference in total running activity of rats treated with either vehicle or 40 mg/kg CORT-treated rats. Values are represented as mean±S.E.M.
(TIF)

Figure S3 **Validation of stress model after 14-day of CORT-injection or chronic mild stress (CMS).** (a) Both the 40 mg/kg CORT injection and CMS increased the plasma CORT level, which acted as stress indicator after 7-day of CORT treatment. (b) The immobility was significantly increased in rats subjected to the CMS and 40 mg/kg CORT treatment when compared with the control. The 40 mg/kg CORT-treated rats showed a longer immobility time. (c) The body weight gain was decreased in the rats with CMS and 40 mg/kg CORT-treated rats. d) The adrenal to body weight ratio was significantly increased in the CMS-treated rats indicating enhanced secretion of stress hormone from the adrenal gland under stress condition. Values are represented as mean±S.E.M. N = 4/group Control: 30 s daily handling; CMS: chronic mild stress according to the protocol described in Table 1; 40 mg/kg CORT: 40 mg/kg CORT-treated rats.
(TIF)

Figure S4 **Locomotor activity was not altered by Ara-c infusion.** (a) The total travel distance in the arena was measured. There was no significant difference among groups. (b) The traveling speed was also not altered by treatment in all treatment groups indicating that locomotor activity was not affected. Values are represented as mean±S.E.M. N = 3–5/group.
(TIF)

Table S1 Chronic Mild Stress (CMS) experimental schedule. The rats were received different stress as described above in different time point continuously for 14 days.
(DOC)

## References

[1] van Praag H, Christie BR, Sejnowski TJ, Gage FH (1999). Running enhances neurogenesis, learning, and long-term potentiation in mice.. Proc Natl Acad Sci U S A.

[2] Redila VA, Christie BR (2006). Exercise-induced changes in dendritic structure and complexity in the adult hippocampal dentate gyrus.. Neuroscience.

[3] Neeper SA, Gomez-Pinilla F, Choi J, Cotman CW (1996). Physical activity increases mRNA for brain-derived neurotrophic factor and nerve growth factor in rat brain.. Brain Res.

[4] McEwen BS, Cameron H, Chao HM, Gould E, Magarinos AM (1993). Adrenal steroids and plasticity of hippocampal neurons: toward an understanding of underlying cellular and molecular mechanisms.. Cell Mol Neurobiol.

[5] Watanabe Y, Gould E, McEwen BS (1992). Stress induces atrophy of apical dendrites of hippocampal CA3 pyramidal neurons.. Brain Res.

[6] Malberg JE, Duman RS (2003). Cell proliferation in adult hippocampus is decreased by inescapable stress: reversal by fluoxetine treatment.. Neuropsychopharmacology.

[7] Schaaf MJ, De Kloet ER, Vreugdenhil E (2000). Corticosterone effects on BDNF expression in the hippocampus. Implications for memory formation.. Stress.

[8] Wong EY, Herbert J (2006). Raised circulating corticosterone inhibits neuronal differentiation of progenitor cells in the adult hippocampus.. Neuroscience.

[9] Gould E, Tanapat P, Hastings NB, Shors TJ (1999). Neurogenesis in adulthood: a possible role in learning.. Trends Cogn Sci.

[10] Gould E, Gross CG (2002). Neurogenesis in adult mammals: some progress and problems.. J Neurosci.

[11] Snyder JS, Hong NS, McDonald RJ, Wojtowicz JM (2005). A role for adult neurogenesis in spatial long-term memory.. Neuroscience.

[12] Santarelli L, Saxe M, Gross C, Surget A, Battaglia F (2003). Requirement of hippocampal neurogenesis for the behavioral effects of antidepressants.. Science.

[13] Lau BW, Yau SY, So KF (2011). Reproduction: a new venue for studying function of adult neurogenesis?. Cell Transplant.

[14] Lau BW, Yau SY, Lee TM, Ching YP, Tang SW (2011). Effect of Corticosterone and Paroxetine on Masculine Mating Behavior: Possible Involvement of Neurogenesis.. J Sex Med.

[15] van Praag H, Shubert T, Zhao C, Gage FH (2005). Exercise enhances learning and hippocampal neurogenesis in aged mice.. J Neurosci.

[16] Saxe MD, Battaglia F, Wang JW, Malleret G, David DJ (2006). Ablation of hippocampal neurogenesis impairs contextual fear conditioning and synaptic plasticity in the dentate gyrus.. Proc Natl Acad Sci U S A.

[17] Wojtowicz JM, Askew ML, Winocur G (2008). The effects of running and of inhibiting adult neurogenesis on learning and memory in rats.. Eur J Neurosci.

[18] Pham K, Nacher J, Hof PR, McEwen BS (2003). Repeated restraint stress suppresses neurogenesis and induces biphasic PSA-NCAM expression in the adult rat dentate gyrus.. Eur J Neurosci.

[19] Vollmayr B, Simonis C, Weber S, Gass P, Henn F (2003). Reduced cell proliferation in the dentate gyrus is not correlated with the development of learned helplessness.. Biol Psychiatry.

[20] Nestler EJ, Gould E, Manji H, Buncan M, Duman RS (2002). Preclinical models: status of basic research in depression.. Biol Psychiatry.

[21] Marks W, Fournier NM, Kalynchuk LE (2009). Repeated exposure to corticosterone increases depression-like behavior in two different versions of the forced swim test without altering nonspecific locomotor activity or muscle strength.. Physiol Behav.

[22] Zhao Y, Ma R, Shen J, Su H, Xing D (2008). A mouse model of depression induced by repeated corticosterone injections.. Eur J Pharmacol.

[23] Woolley CS, Gould E, McEwen BS (1990). Exposure to excess glucocorticoids alters dendritic morphology of adult hippocampal pyramidal neurons.. Brain Res.

[24] Lawlor DA, Hopker SW (2001). The effectiveness of exercise as an intervention in the management of depression: systematic review and meta-regression analysis of randomised controlled trials.. BMJ.

[25] Duman RS, Heninger GR, Nestler EJ (1997). A molecular and cellular theory of depression.. Arch Gen Psychiatry.

[26] Bjornebekk A, Mathe AA, Brene S (2005). The antidepressant effect of running is associated with increased hippocampal cell proliferation.. Int J Neuropsychopharmacol.

[27] Trejo JL, Llorens-Martin MV, Torres-Aleman I (2008). The effects of exercise on spatial learning and anxiety-like behavior are mediated by an IGF-I-dependent mechanism related to hippocampal neurogenesis.. Mol Cell Neurosci.

[28] Airan RD, Meltzer LA, Roy M, Gong Y, Chen H (2007). High-speed imaging reveals neurophysiological links to behavior in an animal model of depression.. Science.

[29] Shors TJ, Miesegaes G, Beylin A, Zhao M, Rydel T (2001). Neurogenesis in the adult is involved in the formation of trace memories.. Nature.

[30] Madsen TM, Kristjansen PE, Bolwig TG, Wortwein G (2003). Arrested neuronal proliferation and impaired hippocampal function following fractionated brain irradiation in the adult rat.. Neuroscience.

[31] Barde YA (1994). Neurotrophins: a family of proteins supporting the survival of neurons.. Prog Clin Biol Res.

[32] Rossi C, Angelucci A, Costantin L, Braschi C, Mazzantini M (2006). Brain-derived neurotrophic factor (BDNF) is required for the enhancement of hippocampal neurogenesis following environmental enrichment.. Eur J Neurosci.

[33] McAllister AK, Katz LC, Lo DC (1999). Neurotrophins and synaptic plasticity. Annu. Rev. Neurosci..

[34] Esposito MS, Piatti VC, Laplagne DA, Morgenstern NA, Ferrari CC (2005). Neuronal differentiation in the adult hippocampus recapitulates embryonic development.. J Neurosci.

[35] Mongiat LA, Esposito MS, Lombardi G, Schinder AF (2009). Reliable activation of immature neurons in the adult hippocampus.. PLoS One.

[36] Wang S, Scott BW, Wojtowicz JM (2000). Heterogenous properties of dentate granule neurons in the adult rat.. J Neurobiol.

[37] Schmidt-Hieber C, Jonas P, Bischofberger J (2004). Enhanced synaptic plasticity in newly generated granule cells of the adult hippocampus.. Nature.

[38] Llado J, Haenggeli C, Maragakis NJ, Snyder EY, Rothstein JD (2004). Neural stem cells protect against glutamate-induced excitotoxicity and promote survival of injured motor neurons through the secretion of neurotrophic factors.. Mol Cell Neurosci.

[39] Lu P, Jones LL, Snyder EY, Tuszynski MH (2003). Neural stem cells constitutively secrete neurotrophic factors and promote extensive host axonal growth after spinal cord injury.. Exp Neurol.

[40] Kempermann G, Gage FH (2002). Genetic determinants of adult hippocampal neurogenesis correlate with acquisition, but not probe trial performance, in the water maze task.. Eur J Neurosci.

[41] Shors TJ, Townsend DA, Zhao M, Kozorovitskiy Y, Gould E (2002). Neurogenesis may relate to some but not all types of hippocampal-dependent learning.. Hippocampus.

[42] McEwen BS, Magarinos AM (2001). Stress and hippocampal plasticity: implications for the pathophysiology of affective disorders.. Hum Psychopharmacol.

[43] Cotman CW, Berchtold NC, Christie LA (2007). Exercise builds brain health: key roles of growth factor cascades and inflammation.. Trends Neurosci.

[44] Berchtold NC, Kesslak JP, Cotman CW (2002). Hippocampal brain-derived neurotrophic factor gene regulation by exercise and the medial septum.. J Neurosci Res.

[45] Stranahan AM, Khalil D, Gould E (2007). Running induces widespread structural alterations in the hippocampus and entorhinal cortex.. Hippocampus.

[46] Rasmusson AM, Shi L, Duman R (2002). Downregulation of BDNF mRNA in the hippocampal dentate gyrus after re-exposure to cues previously associated with footshock.. Neuropsychopharmacology.

[47] Cotman CW, Berchtold NC (2002). Exercise: a behavioral intervention to enhance brain health and plasticity.. Trends Neurosci.

[48] Leuner B, Gould E (2010). Structural plasticity and hippocampal function.. Annu Rev Psychol.

[49] Wang JW, David DJ, Monckton JE, Battaglia F, Hen R (2008). Chronic fluoxetine stimulates maturation and synaptic plasticity of adult-born hippocampal granule cells.. J Neurosci.

[50] Lafenetre P, Leske O, Ma-Hogemeie Z, Haghikia A, Bichler Z (2010). Exercise can rescue recognition memory impairment in a model with reduced adult hippocampal neurogenesis.. Front Behav Neurosci.

[51] Bessa JM, Ferreira D, Melo I, Marques F, Cerqueira JJ (2009). The mood-improving actions of antidepressants do not depend on neurogenesis but are associated with neuronal remodeling.. Mol Psychiatry.

[52] Cerqueira JJ, Mailliet F, Almeida OF, Jay TM, Sousa N (2007). The prefrontal cortex as a key target of the maladaptive response to stress.. J Neurosci.

[53] McEwen BS (2010). Stress, sex, and neural adaptation to a changing environment: mechanisms of neuronal remodeling.. Ann N Y Acad Sci.

[54] Garcia A, Steiner B, Kronenberg G, Bick-Sander A, Kempermann G (2004). Age-dependent expression of glucocorticoid- and mineralocorticoid receptors on neural precursor cell populations in the adult murine hippocampus.. Aging Cell.

[55] Moghaddam B, Bolinao ML, Stein-Behrens B, Sapolsky R (1994). Glucocorticoids mediate the stress-induced extracellular accumulation of glutamate.. Brain Res.

[56] Gould E, McEwen BS, Tanapat P, Galea LA, Fuchs E (1997). Neurogenesis in the dentate gyrus of the adult tree shrew is regulated by psychosocial stress and NMDA receptor activation.. J Neurosci.

[57] Hellsten J, Wennstrom M, Mohapel P, Ekdahl CT, Bengzon J (2002). Electroconvulsive seizures increase hippocampal neurogenesis after chronic corticosterone treatment.. Eur J Neurosci.

[58] Porsolt RD, Bertin A, Jalfre M (1978). “Behavioural despair” in rats and mice: strain differences and the effects of imipramine.. Eur J Pharmacol.

[59] Paxinos G, Watson C (1986). The Rat Brain in Stereotaxic Coordinates..

[60] Lau WM, Qiu G, Helmeste DM, Lee TM, Tang SW (2007). Corticosteroid decreases subventricular zone cell proliferation, which could be reversed by paroxetine.. Restor Neurol Neurosci.

[61] Gould E, Beylin A, Tanapat P, Reeves A, Shors TJ (1999). Learning enhances adult neurogenesis in the hippocampal formation.. Nat Neurosci.

[62] Jung KH, Chu K, Kim M, Jeong SW, Song YM (2004). Continuous cytosine-b-D-arabinofuranoside infusion reduces ectopic granule cells in adult rat hippocampus with attenuation of spontaneous recurrent seizures following pilocarpine-induced status epilepticus.. Eur J Neurosci.

