# Supplementary figures and images for: Hippocampal Neurogenesis and Dendritic Plasticity Support Running-Improved Spatial Learning and Depression-Like Behaviour in Stressed Rats
Source: PLoS One. 2011 Sep 15;6(9):e24263. doi: 10.1371/journal.pone.0024263 (PMC3174166; doi:10.1371/journal.pone.0024263)

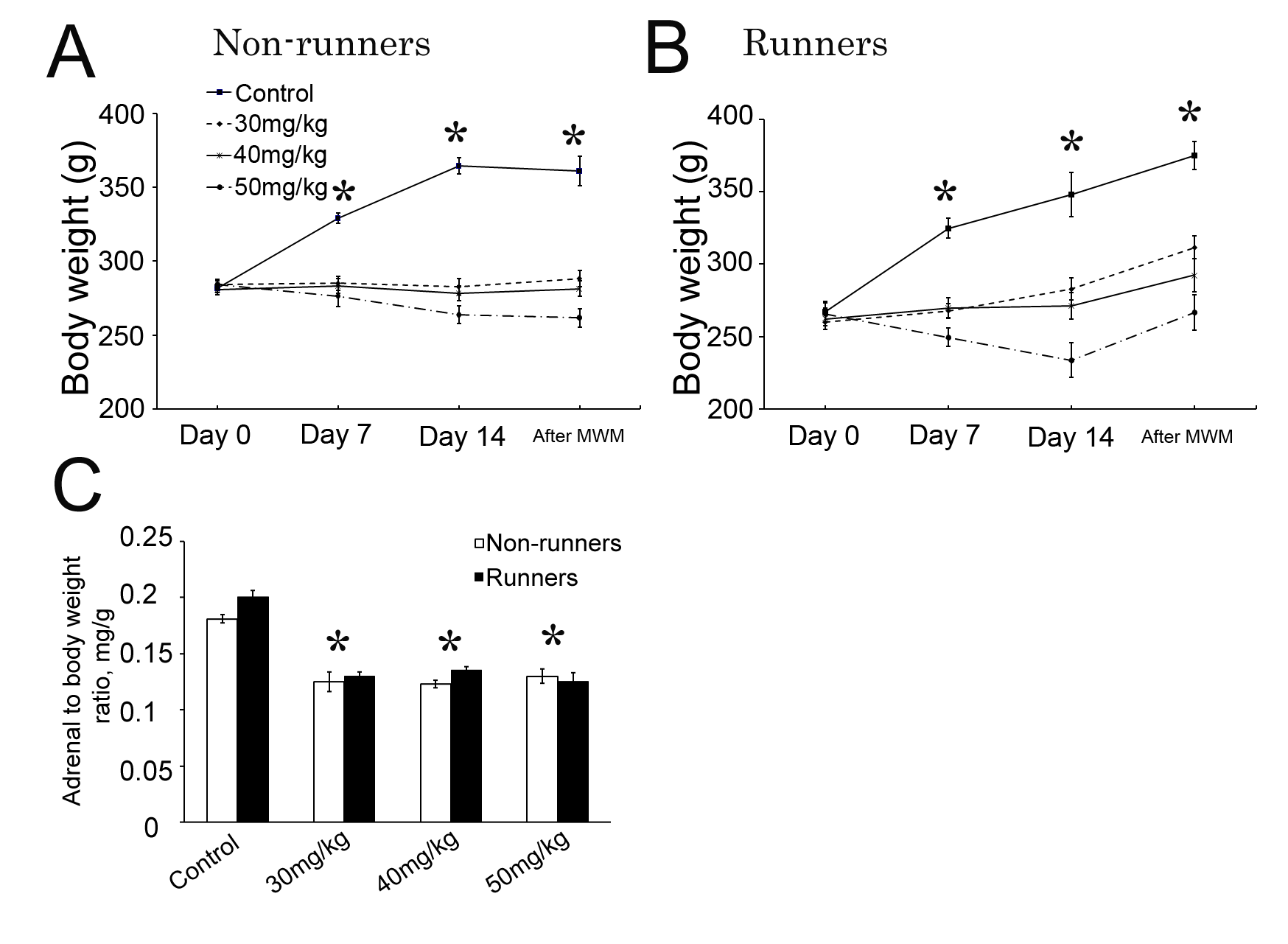

Supplement: Figure S1 — Body weight and adrenal weight change of the rats. a) Non-runners with CORT treatment showed significant decrease in body weight compared to vehicle-treated non-runners. b) Runners with CORT treatments also showed robust decrease in body weight again during the treatment period. C) Adrenal to body weight ratio was significantly decreased by CORT treatment. Values are represented as mean±S.E.M. * p<0.005 compared to the vehicle-treated rats. N = 6–10/group. (TIF) [file pone.0024263.s001.tif]

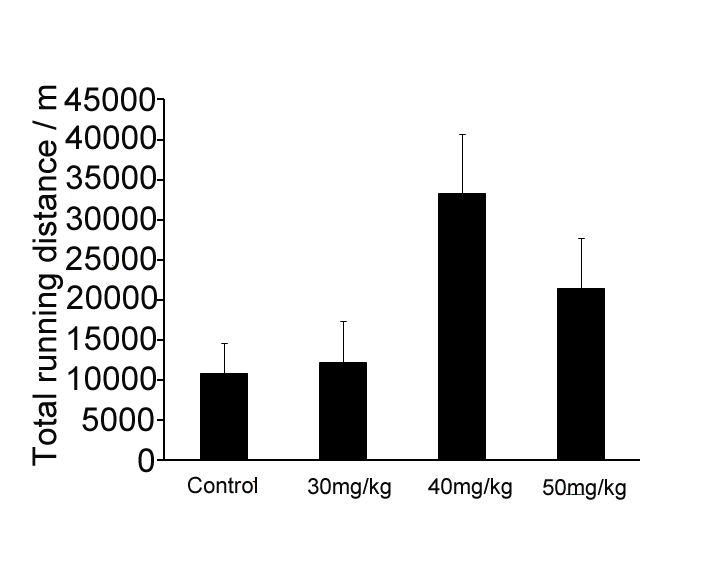

Supplement: Figure S2 — Running activity of the runners. There was no significant difference in total running activity of rats treated with either vehicle or 40 mg/kg CORT-treated rats. Values are represented as mean±S.E.M. (TIF) [file pone.0024263.s002.tif]

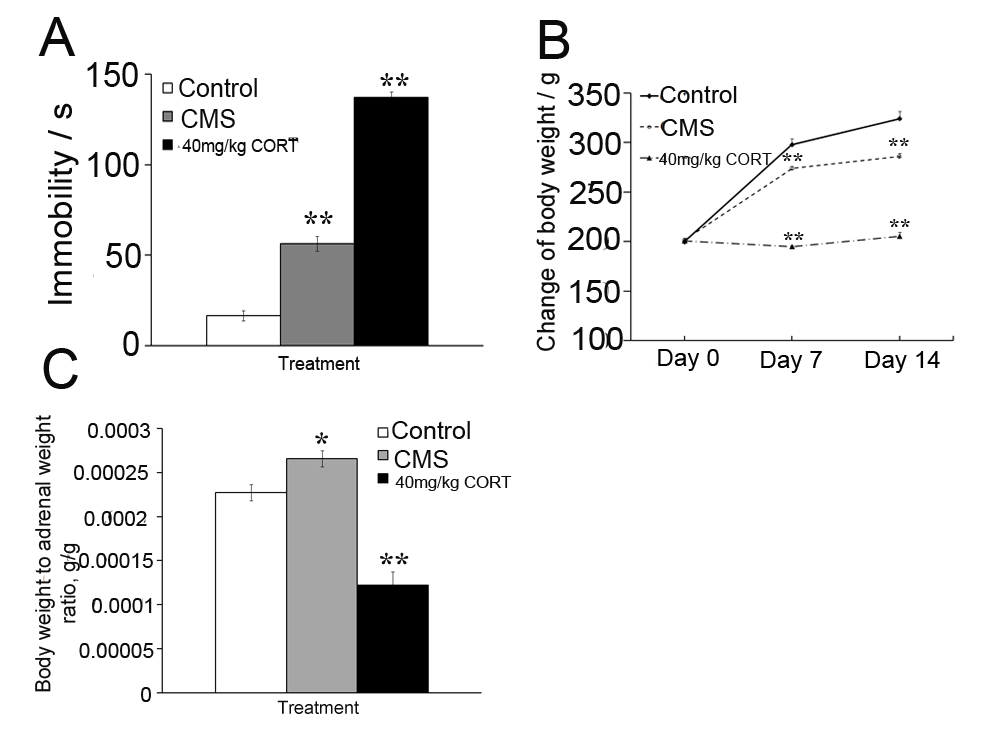

Supplement: Figure S3 — Validation of stress model after 14-day of CORT-injection or chronic mild stress (CMS). (a) Both the 40 mg/kg CORT injection and CMS increased the plasma CORT level, which acted as stress indicator after 7-day of CORT treatment. (b) The immobility was significantly increased in rats subjected to the CMS and 40 mg/kg CORT treatment when compared with the control. The 40 mg/kg CORT-treated rats showed a longer immobility time. (c) The body weight gain was decreased in the rats with CMS and 40 mg/kg CORT-treated rats. d) The adrenal to body weight ratio was significantly increased in the CMS-treated rats indicating enhanced secretion of stress hormone from the adrenal gland under stress condition. Values are represented as mean±S.E.M. N = 4/group Control: 30 s daily handling; CMS: chronic mild stress according to the protocol described in Table 1; 40 mg/kg CORT: 40 mg/kg CORT-treated rats. (TIF) [file pone.0024263.s003.tif]

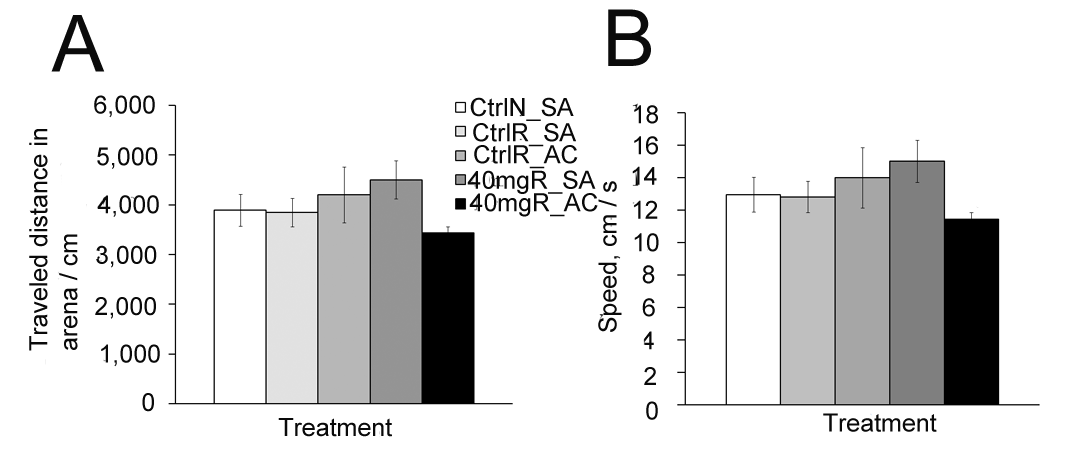

Supplement: Figure S4 — Locomotor activity was not altered by Ara-c infusion. (a) The total travel distance in the arena was measured. There was no significant difference among groups. (b) The traveling speed was also not altered by treatment in all treatment groups indicating that locomotor activity was not affected. Values are represented as mean±S.E.M. N = 3–5/group. (TIF) [file pone.0024263.s004.tif]
