# Supplementary material for: Hippocampal Neurogenesis and Dendritic Plasticity Support Running-Improved Spatial Learning and Depression-Like Behaviour in Stressed Rats
Source: PLoS One. 2011 Sep 15;6(9):e24263. doi: 10.1371/journal.pone.0024263 (PMC3174166; doi:10.1371/journal.pone.0024263)
Supplement: Table S1 — Chronic Mild Stress (CMS) experimental schedule. The rats were received different stress as described above in different time point continuously for 14 days. (DOC) [file pone.0024263.s005.doc]

Supplementary table 1. Chronic Mild Stress (CMS) experimental schedule. The rats were received different stress as described above in different time point continuously for 14 days.

| 1400 Restraint 1hr |
| --- |
| 1100 Crowding and wet bedding 24hr |
| 1500 Shaking 1hr |
| 1000 Overnight isolation |
| 1400 Titled cage (45^0^C) 3hr |
| 1900 Water and food deprivation overnight |
| 1200 Lights of overnight |
| 1400 Restraint 1hr |
| 1500 Overnight isolation |
| 0900 Shaking 1hr |
| 1300 Titled Cage (45^0^C) 3hr |
| 1700 Water and food deprivation overnight |
| 1000 Crowding and wet bedding 24 hr |
| 1500 Restraint 1hr |
